# Food safety and nutritional quality for the prevention of non communicable diseases: the Nutrient, hazard Analysis and Critical Control Point process (NACCP)

**DOI:** 10.1186/s12967-015-0484-2

**Published:** 2015-04-23

**Authors:** Laura Di Renzo, Carmen Colica, Alberto Carraro, Beniamino Cenci Goga, Luigi Tonino Marsella, Roberto Botta, Maria Laura Colombo, Santo Gratteri, Ting Fa Margherita Chang, Maurizio Droli, Francesca Sarlo, Antonino De Lorenzo

**Affiliations:** Division of Clinical Nutrition and Nutrigenomics, Department of Biomedicine and Prevention, University of Rome “Tor Vergata”, Via Montpellier 1, I-00133 Rome, Italy; CNR, ISN UOS of Pharmacology, Department of Pharmacology, University “Magna Graecia”, 88021 Roccelletta di Borgia, (CZ) Italy; Department of Veterinary Medicine, University of Perugia, 06126 Perugia, Italy; Division of Legal medicine and social security, Department of Biomedicine and prevention, University of Rome “Tor Vergata”, 00133 Rome, Italy; Department of Agricultural, Forestry and Food Sciences (DISAFA), University of Turin, 10095 Grugliasco, (TO) Italy; Department of Drug and Science Technology, University of Turin, 10095 Grugliasco, (TO) Italy; Department of Surgery and Medical Science, University “Magna Græcia”, 88100, Germaneto, (CZ) Italy; Department of Civil Engineering and Architecture, University of Udine, 33100, Udine, Italy; Department of Agriculture, University of Naples “Federico II”, 80055, Portici, (NA) Italy; “Nuova Annunziatella” Clinic, 00147, Rome, Italy

**Keywords:** HACCP process, Food safety and security, Total quality management, Chronic non-communicable diseases

## Abstract

**Background:**

The important role of food and nutrition in public health is being increasingly recognized as crucial for its potential impact on health-related quality of life and the economy, both at the societal and individual levels. The prevalence of non-communicable diseases calls for a reformulation of our view of food. The Hazard Analysis and Critical Control Point (HACCP) system, first implemented in the EU with the Directive 43/93/CEE, later replaced by Regulation CE 178/2002 and Regulation CE 852/2004, is the internationally agreed approach for food safety control. Our aim is to develop a new procedure for the assessment of the Nutrient, hazard Analysis and Critical Control Point (NACCP) process, for total quality management (TMQ), and optimize nutritional levels.

**Methods:**

NACCP was based on four general principles: *i*) guarantee of health maintenance; *ii*) evaluate and assure the nutritional quality of food and TMQ; *iii*) give correct information to the consumers; *iv*) ensure an ethical profit. There are three stages for the application of the NACCP process: 1) application of NACCP for quality principles; 2) application of NACCP for health principals; 3) implementation of the NACCP process. The actions are: 1) identification of nutritional markers, which must remain intact throughout the food supply chain; 2) identification of critical control points which must monitored in order to minimize the likelihood of a reduction in quality; *3*) establishment of critical limits to maintain adequate levels of nutrient; *4*) establishment, and implementation of effective monitoring procedures of critical control points; *5*) establishment of corrective actions; *6)* identification of metabolic biomarkers; *7)* evaluation of the effects of food intake, through the application of specific clinical trials; *8)* establishment of procedures for consumer information; *9)* implementation of the Health claim Regulation EU 1924/2006; *10)* starting a training program.

**Results and discussion:**

We calculate the risk assessment as follows: *Risk (R) = probability (P) × damage (D).* The NACCP process considers the entire food supply chain “from farm to consumer”; in each point of the chain it is necessary implement a tight monitoring in order to guarantee optimal nutritional quality.

## Background

We make many decisions in our lives and we weigh the benefits against the drawbacks. Our decisions are based on which benefits are most important to us, and which drawbacks we are willing to accept. Decisions about what we eat are made in the same way; but when it comes to safety, our decisions are usually made more carefully. We need more information to make a wise decision. We need to know where food comes from, what it contains, how the animals were raised or the vegetables grown, and how our government decides which foods are safe for us to eat. Regulations governing food hygiene can be found in numerous early sources such as the Old Testament, and the writing of Confucius, Hinduism, and Islam. Such early writers had at best only a vague conception of the true causes of food-borne illness and many of their prescriptions probably had only a slight effect on its incidence. Even today, despite our increased knowledge, “food-borne disease is perhaps the most widespread health problem in the contemporary world and an important cause of reduced economic productivity” [1]. Deciding whether a food is safe or not is a difficult task. Food can never be proven to be entirely safe nor entirely hazardous. It can only be proven to be hazardous to some degree under certain conditions. While demanding completely safe food is unrealistic, it is possible to have food in which potential hazards have been reduced [2]. For years, safety, i.e. the exclusion or elimination of pathogens from food, has been studied separately from the prevention of spoilage. In most countries the legislation has tended to reinforce this concept. However, from a microbiological-ecological point of view the two areas cannot be distinguished. In spite of considerable efforts, microbiological safety assurance seems as remote as ever, even in advanced countries. Death, suffering, economic losses and civil claims on behalf of victims of food-borne diseases are matched by the economic losses caused by food spoilage [3].

Moreover, semantics used by food safety scientists sometimes can be deceiving. For instance the English sense of ‘to control’ in food science is to assure good quality and safety. On the other hand the Italian word “controllo” or the German “Kontrolle” correspond to the English words ‘inspection’ or ‘monitoring’. Therefore, among scientists, the English word ‘control’ has been widely adopted to mean ‘management’ as it is used in medicine, e.g. the management/control of pain. The right strategy is the so-called ‘forward control to assure food safety’. In the past the control of safety and quality of food was achieved, although we should use the sentence: ‘expected to be achieved’, by the retrospective, repressive or backward control system. This consists of getting samples after food has entered the food supply chain, examine for pathogens, spoilage or marker microorganisms and then taking the appropriate actions. This system fails for two reasons. First of all the retrospective approach is, indeed, an inspection, which can only measure an effect without identifying a mechanism and hence can never lead to management of risk. Secondly the sample numbers should be chosen randomly and in consideration of the *poisson* distribution. This requires an incredibly high number of samples to be tested and the, at that point useless, feedback to the manufacturer or supplier. The intervention approach, instead, extended all along the food production, distribution and storage lines, leads to adequate consumer protection. This includes drawing up and adhering to what have been termed “codes of good manufacturing and distributing practices”. The European Union Directive 178/2002 and the resulting regulations, commonly known as “the food hygiene package”, and the more recent regulations laying down detailed rules for the organization of official controls on products of animal origin intended for human consumption, indicate the stages to be applied. They include the design of ways for the elimination of all identified critical sites and practices, relying on holistic quantitative risk analysis (the HACCP – hazard analysis and critical control point – concept); the implementation of the required intervention steps all along the production, distribution and culinary preparation lines (the LISA – longitudinally integrated safety assurance – concept); and the meticulous codification of procedures to be followed throughout (the GMDPs – good manufacturing and distribution practices – concept). Moreover, in the new cited proposal it is postulated that the general hygiene rules be extended so as to cover hygiene at the farm level. In doing so, European Community legislation on food hygiene will be provided with an instrument that covers the entire food chain, from farm to table. To achieve the required level of hygiene at farm level, it is suggested that possible hazards occurring in primary production and methods to control such hazards shall be addressed in guides to good practice.

Although the food safety system proposed at the level of primary production is risk based, a formal implementation of the HACCP system is not foreseen. Such a system could possibly be introduced at a later stage when experience with the new hygiene rules demonstrates that it can be practically applied at the primary production level.

Food contains natural chemicals and it can come into contact with many natural and artificial substances during harvest, production, processing and preparation. They include microorganisms, chemicals (either naturally present or produced by processing), environmental contaminants and pesticides. Since the chance of being harmed by these potential hazards is called risk, risk analysis might be better termed as the science of food safety, because risk management is an essential part of it. A good illustration of two aspects of risk analysis refers to the tale of the Sword of Damocles. The size of the danger is determined both by the weight and sharpness of the sword (the hazard) and by the strength of the rope holding it (the risk) [3].

An important debate is ongoing at the national and international levels concerning the role precaution should play in guiding policy decisions. Food safety discussions reflect the need to find a better balance between reaping the benefits of technology and innovation on one hand, and avoiding or minimizing the risk of unacceptable adverse side effects of technological progress on the other. Experience with unexpected adverse effects of new chemicals over the past half-century has led to growing support for application of the so-called “precautionary principle”. The precautionary approach calls for developing better mechanisms for anticipating adverse side-effects of new technologies, and for reviewing technologies more thoroughly, exploring alternative ways for reaping benefits while minimizing adverse collateral effects, before any major innovation is widely adopted [4]. The essence of precautionary risk assessment is to treat scientific questions scientifically. Often, in food safety risk analysis, science is used politically. A precautionary risk assessment takes a broader approach, defining a full array of risk-related questions needing answers [4]. The conceptual distinction between risk assessment (understanding) and risk management (action) is useful for various important purposes, such as insulating scientific activity from political pressure and maintaining the analytic distinction between the magnitude of a risk and the cost of coping with it. For the purposes of improving decision-relevant understanding of risk and making that understanding more widely accepted, however, a rigid distinction of this sort does not provide the most helpful conceptual framework. The reason, in brief, is that the analytical activities generally considered to constitute risk assessment are not sufficient by themselves to provide the needed understanding.

The globalization of trade, which has contributed to food availability and diversification through the world, has also increased the chances that food produced in one place will affect the diet and therefore the health status of people living in another [5]. Over the last few decades, the problem of food-borne illness has increased significantly, enough to be the greatest public health problem in industrialized countries, where health problem related to food consumption are linked to two main factors: food safety and nutritional risk [6].

The spread of chronic non-communicable diseases (CNCDs), has forced us to reconsider our viewpoint on food security. CNCDs are non-infectious and non-transmissible diseases, of long duration and slow progression, including obesity, cardiovascular diseases, diabetes, chronic kidney disease, osteoporosis, sarcopenia, Alzheimer’s disease and cancers.

As referred in the WHO 2010 report [7], the importance of establishing preventive health strategies has been widely acknowledged [8]. The prevalence of CNCDs is rising rapidly and WHO projections show that CNCDs deaths are projected to increase by 15% globally between 2010 and 2020 (to 44 million deaths) [7,8].

The effects of dietary compounds on CNCDs are currently under investigation and are directing traditional nutritional counseling towards a more complex approach based on gene expression modulated by food. Considering these “food related” pathologies together, the burden of disease in western countries is impressive [9]. Moreover, in the post genomic era, food is considered not only a reservoir of macronutrients, vital in the maintenance of cellular metabolism, but also a major factor capable of determining the quality of health. The close relationship that exists between micronutrients and gene expression may underlie the pathophysiologic phenomena or, conversely, may represent an early target in delaying the onset of CNCDs [10]. Nutrition is undoubtedly a major modifiable determinant of disease. Over the last decades, the interest in evidence-based health care has grown considerably. In the same time period, the economic evaluation of health care technologies has been instituted [11].

A food supply chain is a network of related business firms input producers, farms, food processors, distributors, wholesalers, retailers and consumers through which agri-food products move from production through to consumption, including pre-production and post-consumption activities [12].

However, at present HACCP programs and good manufacturing practices (GMP) are mainly used to manage hazards in foods. While HACCP has proven to be very effective for the control of food safety [13], it must be acknowledged that it is designed on the basis of known hazards, and that potential future risks are not necessarily taken into account. Moreover, the maintenance of nutrients all along the food chain is not considered.

On the other hand, the food industry has proceeded to tackle nutrition and health-associated challenges in two complementary ways: *i*) by removing or replacing unhealthy ingredients; *ii*) by incorporating healthy or health-promoting ingredients and bioactive compounds into new products (e.g. functional foods).

Nowadays, we need not only safe food (already guaranteed by the HACCP process), but food that can help the consumer maintain a good state of health. Consumers are very careful about food quality, not only in terms of hygiene standards, but also from the nutritional point of view. Moreover, the concept that environmental conditions and physiological factors may modify the amounts of phytochemicals present in vegetables and fruits is widely accepted, together with the theory that crop management strategies are capable of modifying phytochemical production [14]. Therefore, the effects of mineral nutrition, soil composition and water content on the production of phytochemicals have been considered in the development of different fertilization strategies, efficient water management and newer techniques such as grafting. The contents of health-promoting compounds in vegetables and fruits depend also quantitatively and qualitatively on their genetic makeup. Thus, conventional breeding and genetic modification have been developed as new methodologies to enhance the nutritional properties of plants. The amount of phytochemicals can be modulated by specific crop protection measures [15], but it can also affect the safety of food, due to the presence of multi-residues of agrochemicals.

In this contest, the aim of the present study is to develop a new procedure for the assessment of the Nutrient, hazard Analysis and Critical Control Point (NACCP) process, to ensure nutritional quality of food throughout all stages of production, in order to define the impact on human health.

The NACCP process aims to evaluate and guarantee total quality management (TMQ) in the maintenance of high nutritional levels with a consequent positive impact on consumer’s health.

NACCP is based on the principle that the food issue must be dealt with using a “holistic” approach, targeting both safety and nutritional aspects. Increased awareness of both elements (safety and nutrition) generally encourages healthy eating and good habits, and prevents acute and chronic diseases [16]. This double intervention is innovative and no other similar processes have been adopted in Italy or elsewhere. This proposition introduces a new tool in the management of international safety, quality and human health.

## Methods

In a broad terms there are three stages to the application of the NACCP process:

- Stage 1 is the application of NACCP for quality principles.
- Stage 2 is the application of NACCP for health principals.
- Stage 3 is the implementation of NACCP process.

Each of these stages will be considered in the context of making NACCP really work in practice.

### Stage 1- Application of NACCP for quality principles

**Figure 1:**
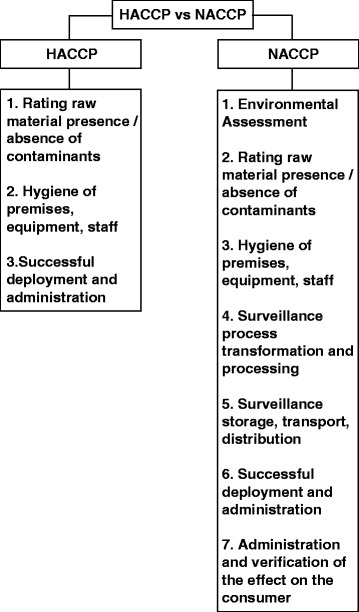
NACCP Process as evolution of HACCP System for nutritional quality assessment. HACCP: Hazard Analysis of Critical Control Point (Reg. EU 852/2004); NACCP: Nutrient, hazard Analysis and Critical Control Point Process.

To maintain nutritional quality it is necessary to act on all aspects of the food supply chain. The NACCP process, through the tracing of a nutritional biomarker, represents the conceptual and scientific evolution of the HACCP system, with nutritional quality of food in mind and the consumer’s health status as its main objective [17,18] (Figure 1).

**Figure 2:**
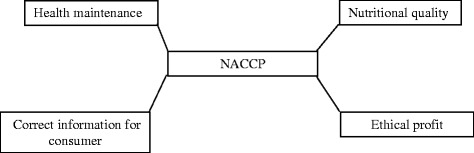
The four principles of Nutrient & Hazard Analysis and Critical Control Point Process.

Behind the NACCP process, there are four general principles in order to ensure: *i*) health maintenance; *ii*) nutritional quality assurance; *iii*) correct information for the consumers; *iv*) ethical profit (Figure 2).

**Figure 3:**
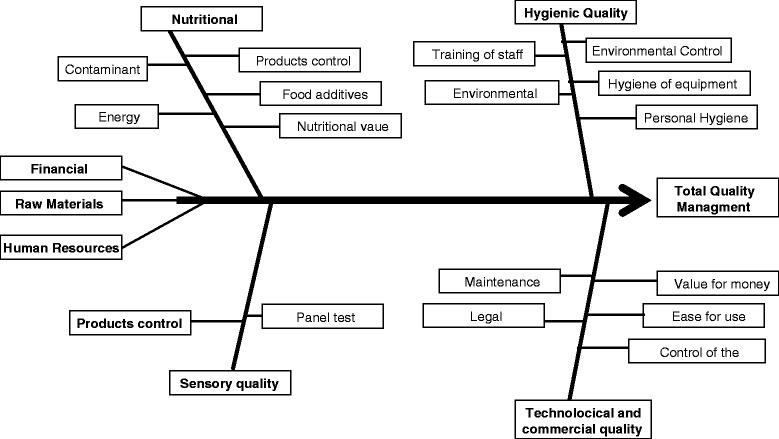
The NACCP concept.

Before food arrives on the table, it must follow a path along which it undergoes many transformations, which may lead to depletion of the nutrient content thus rendering it irrelevant at the health level. At present, therefore, the main objective is to be able to trace not only the food, but the nutrient of interest, which must be kept intact along the entire production chain to guarantee a real health benefit to the consumer (Figure 3).

On the other hand, it is important to guarantee the quality of those foods such as fruit, vegetables, fish and meat that have not undergone industrial transformation, in order to provide a health-giving product to the consumer without causing harm.

### Stage 2- Application of NACCP for health principles

**Figure 4:**
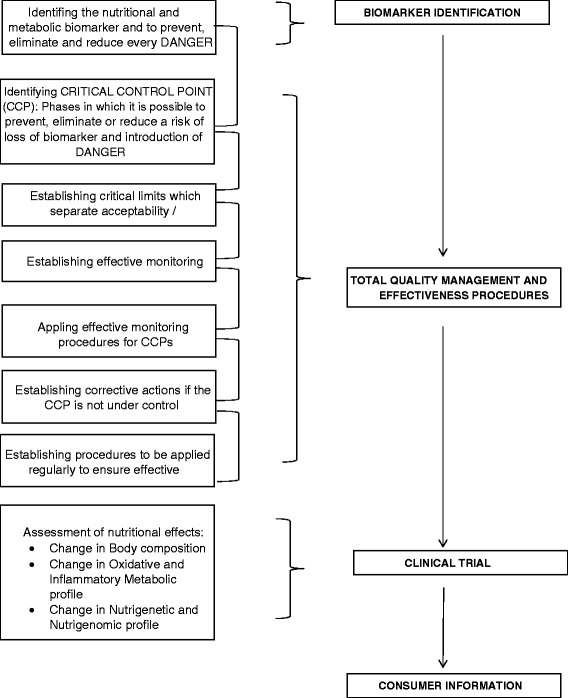
Phases of NACCP process.

As highlighted in Figure 4, the actions of the NACCP process are envisaged to ensure high quality, nutritional food for the maintenance of good health through the prevention of CNCDs.

To make NACCP work in practice, the following actions must be adhered to: 1) identification of the nutritional marker (macronutrient, micronutrient, mineral salts, vitamins, antioxidants, dietary fiber), which must remain unchanged throughout the chain of production; 2) identification of critical control points of the food chain (area of production, technologies of cultivation and breeding, processing, heat treatment, transport, distribution and administration), which must be regulated in order to minimize the likelihood of a reduction in quality; 3) establishment, within the critical control points, of critical limits to maintain adequate levels of nutrient; 4) establishment, and implementation of effective monitoring procedures of critical control points; 5) establishment of the corrective actions to be taken when the monitoring indicates a non-conformity of a critical point; 6) identification of metabolic biomarkers; 7) evaluation of the effects of food intake, through the application of specific clinical trials; 8) establishment of procedures regarding consumer information; 9) implementation of the Health claim Regulation EU 1924/2006; 10) starting a training program.

The biological validity of the nutrient must be maintained throughout all the cited steps so that the food reaching the end consumer is both safe and physiologically significant. While the first five actions of the NACCP process have an ameliorative and preparatory function, for the nutritional quality maintenance of the food, the six and seven actions identify a biomarkers which allow future examination of the effect of a single nutrient on the consumer’s health, the last three actions aimed to educate and inform.

The NACCP process actions are described as follows:

- Action 1: Identification of a nutritional biomarker.

Conformation with all steps of food supply chain should preserve the quality and amount of a selected nutrient. Nutrients, as well as ensuring the vitality of metabolic functions, affect the enzymes involved in physiological processes, effectively determining whether health is good or bad. According to Regulation CE 178/2002 a nutrient can be found into the following categories: *i*) nutrient: a food constituent in a form and at a level that helps support life; *ii*) dietary supplement: a product that contains one or more of the following dietary ingredients: vitamin, mineral, amino acid (protein) and also includes concentrates, constituents, extracts or metabolites of those compounds; *iii*) a nutraceutical: any nontoxic food component that has scientifically proven health benefits, including disease treatment and prevention.

Young defined a nutrient as “a fully characterized (physical, chemical, physiological) constituent of a diet, that serves as a significant energy yielding substrate, or a precursor for the synthesis of macromolecules or of other components needed for normal cell differentiation, growth, renewal, repair, defense and/or maintenance or a required signaling molecule, cofactor or determinant of normal molecular structure/function and/or promoter of cell and organ integrity” [19]. Therefore some functions of a nutrient can be those of signaling molecules [20] and substrate for macromolecules [21-23]. The nutrient can also modify molecular structures and promote assembly of mechanistic structures.

Food is more than metabolic fuel. There is good evidence that nutrients influence gene expression and there is an interdependence of morphologic expression of an organism with its genetic sequence and to its surrounding environment, including diet and life-style. Nevertheless, metabolism is dynamic and it changes in relation to the variations determined by environmental factors [24].

Selection of a nutritional biomarker with a potentially beneficial effect on the consumer’s health and the determination of its bioavailability appear to be of primary interest in the evaluation of the function and the effects arising from the consumption of a nutrient [25].

For the selection of a nutritional biomarker it is important to keep in mind:

1. viability of identification, quantification and tracing of a selected nutritional biomarker. Moreover, the nutritional biomarker must remain intact throughout the entire food supply chain. Cellular events can modify response to bioactive food components (BFC), and on the other hand BFC can modify cellular events. This defines real nutritional homeostasis [26];
2. execution of physical-chemical and qualitative (bromatological and microbiologcal) analysis to ensure that food contains the specific nutrient biomarker that interacts in a specific salutary way on the physiological functions of the organism;
3. demonstration that the nutrient could determine a disease state, and may be toxic over an established threshold level [19,27]. Therefore, to prevent that nutrient becoming harmful to health awareness of its unique chemical structure and the dose to be administered is necessary. The analytical evaluations include: *i*) chemical characteristics: data relating to the chemical-nutritional food properties such as macro and micronutrients, minerals, fiber, vitamins, antioxidants during the processing stages; *ii*) physical characteristics: data relating to processes, such as any heat processing, pasteurization, killing organisms during thermal maturation and ripening; *iii*) microbiological characteristics: data on food symbiotic microorganisms, or probiotics that confer special properties (presence of particular strains of lactic acid-bacteria or molds and yeasts in dairy products).

- Actions 2–5 Identification of Critical Control Point, Critical Limit, Corrective Actions.

Over the years, several scientific and technical recognitions have been instrumental in developing and forming principles and techniques to achieve acceptable food safety in certain conditions. According to Raspor [24], today the principal aim of food safety is to guarantee consumer’s health, through the absence of chemical, physical and biological contamination in food. For example, with regard to genetic toxicology, basic foodstuffs have generally been considered safe, but questions of safety, and particularly of the long-term effects of ingesting mutagenic/carcinogenic substances in food, have arisen with the development of food processing and the use of chemicals to improve the quality, palatability and shelf-life of food products. The intake of mutagens in the regular diet may exceed by far the amount taken in from industrial sources. The human population consumes about 10 tons (dry weight) of food by the age of 50 [28]. Food-related genotoxins are important because of the extent of the population exposed and because habit or custom leads to the frequent intake of certain foods. In vitro short-term mutagenicity assays have revealed that a number of naturally occurring constituents of foods and of compounds formed during processing are mutagenic [29]. Flavonoids, furans and some mycotoxins are among the naturally occurring constituents that have been shown to be mutagenic. Mutagens have also been found in certain classes of food and environmental contaminants (e.g. pesticides, packaging components, solvents) and additives (e.g. some colorings and flavorings) and among products formed during heating, irradiation, smoking, curing, solvent extraction, fumigation and storage. Ideally, testing for possible mutagenic effects requires in vivo studies, but at the present time, in vitro tests provide the only practical means of screening for mutagens in the large variety of food consumed and of studying factors that modify the mutagenic activity of food constituents. Such tests may also be used to assess how food processing methods may be modified to reduce the generation of mutagens.

Human’s food is obtained from a large number of sources and species and is characterized by its great variety. Before it is consumed it is subjected to various treatments. The toxicology of food is, thus, a complex problem. Society has recognized for generations that certain potential foodstuffs, such as some plants and fishes, are acutely toxic and social taboos prevent their consumption. However, acute toxic incidents cannot be relied upon to discourage the ingestion of food containing genetic toxins, since the latent period of the genetic effect may be prolonged, possibly stretching over several generations.

The HACCP system represents the most intelligible example of this development [30]. The application of all seven principles: *i.* Conduct to hazard analysis; *ii.* Identify critical control point; *iii.* Establish critical limits; *iv.* Monitor CCP; *v.* Establish corrective action; *vi.* Verification System; *vii.* Record keeping, is a prerequisite for the success of the NACCP process.

Each step of the food supply chain has its own HACCP system distinct from prior and subsequent steps. In order to tackle the existing barriers in implementing and maintaining food safety system [31,32], it is necessary to unify total quality management, through compliance with good manufacturing practice. A critical control point, is represented by each step of food chain and it should be considerate a point in which nutritional biomarker can be reduced. Moreover, for every single step or sub-process of the food chain, a hazard analysis has to be performed [33].

- Actions 6 and 7: Identification of metabolic biomarkers and evaluation of nutrients on consumer’s health.

It is important to identify a measurable metabolic biomarker that is modulated in some way by the nutrient, and that reflects the nutritional effects of the “active” ingredient, or combination of food ingredients. Specific metabolic biomarkers, defined as molecules or groups of molecules whose simple presence is an indicator of a problem, state or condition, are required to understand the threshold value and eventually evaluate an excess (or deficit) of nutrient metabolism products. Since a single metabolic biomarker is often insufficient for assessment of metabolic disease, measurement of amounts of specific metabolic intermediates are frequently needed [27,34].

Since imbalances between the concentrations of metabolites, and not the appearance or disappearance of any single intermediate, forms the basis for metabolic disease, only quantitative and comprehensive metabolite measurements can identify metabolic imbalance [27].

An ideal metabolic biomarker: *i*) should respond sensitively, specifically and predictably to changes in the concentration and/or supply of the micronutrient; *ii*) should be amenable to objective and reproducible measurement both of form and quantity which should reliably reflect a change in the target tissue that has a direct effect on health; *iii*) should be in a measurable dose–response relationship [35].

The chief classes of metabolic biomarkers are: *i*) cholesterol, high-and low-density lipoproteins (HDL, LDL), oxidized LDL, triglycerides; *ii*) transaminases, glucose, and insulin; *iii*) homocysteine, fibrinogen, C-reactive protein, erythrocyte sedimentation rate, and proinflammatory cytokines; *iv*) albumin, prealbumin, and retinol binding protein.

Due to the innate complexity of human biology, compounded by a myriad of social and environmental factors that are crucial determinates of health, we propose experimental systemic approaches which permit integration of diverse data types to create an actionable model to verify the effects of foods. In fact, a modern approach to medicine and health requires that enormous amounts of information, such as social, medical, clinical, molecular, cellular, genetic, demographic, and environmental data, need be deciphered and integrated into a model that includes network interactions and integrations at many levels, relaying relevant biological and environmental information [36].

The analyses for investigating the effect of food must be conducted at various levels to identify the degree of interaction of the nutrient with the human body and its possible effects.

We define three levels of diagnostic investigation to verify any nutritional effects.

1. Assessment of nutritional status by: a) family and individual history-taking; b) anthropometry (weight, height, circumferences, skinfolds); c) body composition analysis: determination of water compartments, such as Total Body Water (TBW), Extra Cellular Water (ECW), Intra Cellular Water (ICW), Body Cellular Mass (BCM), Body Cellular Mass Index (BCMI); Evaluation of lean body mass, fat and bone mineralization; d) Nutritional survey of dietary habits (Indali, Simplified Nutritional Appetite Questionnaire, i.e. SNAQ questionnaire); e) Functional evaluation: measurement of blood pressure and heart rate; assessment of physical activity (motor questionnaire, tests of strength and muscle power); assessment of quality of life (questionnaire of quality of life);
2. Clinical and biochemical assessment: lipid profile, carbohydrate profile, liver profile, oxidative and inflammatory profile. Evaluation of inflammation and oxidative stress markers of DNA damage, oxidative stress marker, damage to lipids, inflammatory profile, etc. by using innovative analytical techniques (microarrays, real time PCR).
3. Nutrigenetic and nutrigenomic assessment. As gene activation may be the result of various combinations of lifestyle and genetic factors, it is necessary to evaluate and manage both types of information. Moreover, given that significant evidence exists demonstrating the influence of genetic variation on dietary responses in human, an option in diseases prevention may be to use nutritional agents to modulate the biological results stemming from genetic variation [37]. In fact, many nutrients selectively alter gene expression through transcription factor systems that regulate the activation of specific sets of genes in different tissues and under different environmental conditions. Various nutrients bind to or in some way directly activate specific transcription factors and other nutrients alter the oxidation reduction status of the cell to indirectly influence transcription factor activity [38,39].

According to this evidence, practical application of nutrigenomics requires:

1. identification of the genes and proteins expressed differentially in health and disease that are modifiable by nutrients;
2. identification of genes, proteins, and metabolites that are influenced by specific nutrients known to be beneficial or harmful;
3. identification of genetic variations that alter the nutrient– gene interactions in applications 1 and 2.

Subsequent nutritional interventions may help detect changes in gene expression related to intake of a particular food. Up-regulation or down-regulation of each gene in relation to baseline can then be determined, in relation to all administered interventions.

The three levels of diagnostic investigation can be grouped by macro-ranging biomedical analysis to analyze individual physiological aspects and analyzed together to formulate a grade of interaction (GI) of the nutrient.

We have defined the degree of interaction as “*effective nutraceutical food*”, evaluated through the different levels of biomedical analysis.

- Actions 8 and 9: Implementation of Health Claim regulation and Information for Consumers.

There is general consensus among scientists, consumers, authorities as well as industry that health claims on (functional) foods must be scientifically substantiated. This is in the interest of all stakeholders and contributes to fair trade. Several important developments have been made within the European Union; these cover scientific as well as regulatory aspects.

Food products that boast nutrition and health-promoting properties are becoming increasingly popular in the EU market. A nutrition claim states or implies that a food has beneficial nutritional properties such as “low fat”, “no added sugar” or “high in fiber”. Any statement given on the label, or used for advertising or commercial purposes, whereby the consumption of a particular food can be beneficial to health, is considered a health claim, such as claims that a food can help to strengthen the body’s natural defenses or improve learning ability [40].

Nutrient profiling is the classification of foods for specific purposes based on their nutrient composition. The establishment of nutrient profiles is essentially a way of classifying foods based on their nutrient content to determine the permissibility of a food to bear a claim. They are intended to prevent claims from masking the overall nutritional profile of a food and should be based on generally accepted scientific evidence relative to the relationship between diet and health. On request from the European Commission, who has the body responsible for their establishment, EFSA has provided scientific guidance on the setting of nutrient profiles within the context of the regulations taking into account the role of food groups within the diet [41]. The two key objectives of the claim are: i) to ensure that consumers are not misled with regard to claims made on or about food; ii) to facilitate cross-border trade within the EU. Nutrition claims impart information regarding the amounts of energy, nutrients and/or other substances.

In the same way as the evaluation of the effect of nutrition on the final consumer is the main goal of the whole system, so too is the proposal of a nutritional claim. The ability to verify the effect of the food and then the nutritional biomarker on human health, may allow the formulation of nutrition labeling as required by EC Regulation 1924/2006. As regards to the regulatory aspects, in December 2006 the EU adopted Regulation 1924/2006 on nutrition and health claims made on foods [Reg. EC 1924/2006]. The general objective of Regulation 1924/2006 is to harmonize the national rules on nutrition and health claims. Nutrition claims are claims that state, suggest or imply that a food has particular beneficial nutritional properties due to the energy it provides or the nutrients it contains [42]. The regulation lays down further restrictions on the use of nutrition and health claims through nutrient profiling. However, according to a recent publication, most proposed nutrition and health claims were negatively assessed by the European Food Safety Authority (EFSA), based on the quality of scientific substantiation, due to usage of scientific methods on which no consensus has been reached and the differences in expectations and requirements [43].

A nutrition claim states or implies that a food has beneficial nutritional properties. Foods with health claims may have an impact on dietary behavior; adoption of nutrient profiles might also stimulate the development of products with an improved nutritional composition by the food industry and as such food reformulation can contribute to public health [5].

- Action 10: Training program.

One of the actions that are necessary for the success of the process is undoubtedly the organization of a trained team of experts capable of initiating and implementing NACCP. A successful NACCP team must have a clear understanding of the importance of identifying both the hazards and nutritional biomarkers, as well as the critical point that require monitoring. Therefore, the selection of a quality team member should be based on knowledge of raw material, products, processes, hazards, molecular biology, food chemistry, nutritional quality, clinical nutrition. The team must be prepared with in-depth training in the principles of NACCP and of the special skills and topics which underlie the application of these principles. It is necessary that the team has a complete knowledge of the NACCP vision, and has a precise understanding of the the initial actions to undertake as well as a clear perception of the end result.

### Stage 3- Implementation of NACCP process

The production of each food can be controlled throughout the NACCP procedure, but not all foods have the same degree of interaction with the human body, which ensure beneficial effects on human health or prevent certain types of diseases.

Corresponding with this vision for nutritional safety, every step can be linked to create a unique good practice approach, called Good Nutritional Practices (GNP). In the NACCP system, the GNP are the critical control points, because the adherence to good practice at every single step of the food production process guarantees the presence of nutritional biomarkers and therefore total nutritional quality.

As described by Raspor [33], good practices concern: *i*) *agriculture*: as agronomic cultivars; the conditions for the growth and reproduction of the plant for the uptake of nutrients from the soil; the type of fertilizer needed for the development of plants; cultivation techniques (conventional, organic, biodynamic, homeodynamic); animal breeds and any mutations that can produce specific phenotypic characteristics; clinical and veterinary health status of animal; genetic and growth capacity; type of relaying; type of power supply. ii) *environment*: such as climatic and chemical conditions of the soil; *iii*) *manufacturing-retail*: the techniques of food processing represent important steps in the food production chain, since during processing, a large quantity of nutrients or other substances with potential beneficial effects on human health may be lost; *iv*) *laboratory*: such as qualitative systems governing organizational process, monitoring, recording and reporting; *v*) *hygiene*: such as practical procedures that return the processing environment to its original condition (disinfection or sanitation programs) and maintain food in optimal storage conditions; *vi*) *storage*, *transport* and *distribution*: these are of the utmost importance throughout the process, as all the nutritional characteristics derived from previous phases must be preserved and arrive intact to the consumer; *vii*) *housekeeping*: the selection of the principles and techniques of food storage and preparation at home directly carried out by the consumer.

The NACCP process evaluates the risk of loss of the nutritional biomarker as a result of nutritional practices at every step of the food supply chain. To understand in full the possibility of loss of a nutritional biomarker during a particular phase, it is necessary calculate the risk for the event. The criteria of analysis and evaluation are based on objective studies of the critical issues, identified by evaluating the actual likelihood of occurrence of an event directly attributable to the critical issues encountered. This probability is related to the gravity of the damage resulting from the occurrence event.

It is therefore necessary to verify any critical point in primary production, food practices and facilities, storage, retail, distribution and household activities.

If results from the monitoring of critical control points indicate that the process is out of control, corrective actions, tailored to the severity of the risk, have to be undertaken.

Corrective action is necessary when the parameter monitored has exceeded the specified critical point, and moreover demonstrates the likelihood that the quality of food is affected or even lost. Therefore, it is necessary to implement appropriate corrective actions to regain control of the condition and return within threshold values of parameters within which preservation of the nutritional quality of the food is guaranteed.

Corrective actions can be classified into two categories: *i*) preventive actions; *ii*) controls identifying finished products not meeting the terms of nutritional quality.

Only when all the critical points of the process will be under control it will be possible start to the assessment of the nutrient health effects through actions n. 6 and 7 described in the Stage 2.

## Results and discussion

We describe a scale of probability of occurrence of an event resulting from the critical issues of any given food supply chain. A scale of the damage caused to that nutritional biomarker as a result of the occurrence of a given event during a critical step, is associated with this scale of probability.

The scale of the probability of the occurrence of a dangerous event and those related to injury have the same quantitative definition so that the determination of the risk factor as shown in Tables 1 and 2, is homogeneous.

**Table 1:** **Scale of occurrence of an event “P”**

***Value***	***Level***	***Definition***
4	High probably	There is a direct correlation between the phase detected and the occurrence of the damage in terms of loss of nutritional biomarker; it is usually a phase that leads to loss of nutrients.
3	Probably	The phase detected can cause damage, even if not in automatic mode or direct.
2	Unlikely	The phase detected can cause loss of nutritional biomarker to the simultaneous occurrence of certain conditions.
1	Improbable	The phase detected can cause damage to a combination of several independent events unlikely.

**Table 2:** **Scale of entity of damage “D”**

***Value***	***Level***	***Definition***
4	Very serious	Total loss of nutritional biomarker and impoverishment quality of the food. Possible creation of harmful molecules to human health.
3	Serious	Almost total loss of biomarker nutritional and impoverishment quality of the food.
2	Medium	Partial loss of the biomarker nutritional and impoverishment quality of the food.
1	Soft	Reduced loss of the biomarker nutritional.

Once the injury and the probability are defined, the risk is automatically determined by the formula:

$$ Risk(R)= probability\kern0.5em (P)\times damage\kern0.5em (D) $$

We obtained an Evaluation Risk Matrix where the abscissae represents the severity of the damage expected and the ordinate the probability of its occurrence (Table 3).

**Table 3:** **Evaluation Risk Matrix**

***Probability***	***4***	4	8	12	16
	***3***	3	6	9	12
	***2***	2	4	6	8
	***1***	1	2	3	4
		***1***	***2***	***3***	***4***
	***D-damage***				

Risks that may cause the most serious damage in the context of a matrix of risk evaluation, as shown in Table 3, are found in the top right hand squares (high probability, serious damage); slight damage and negligible probability on the other hand are found in the positions closest to the origin of the axes, with the whole series of intermediate positions easily identifiable between the two. This representation is a starting point for the definition of priorities and the schedule of prevention for preserving the nutritional biomarker.

The numerical evaluation of the level of risk “R” requires the implementation, prevention and protection of measures in relation to the risk assessment, as shown in Table 4.

**Table 4:** **Risk identification**

**R > 8**	Corrective actions to be implemented immediately
**4 ≤ R ≥8**	Corrective actions to be implemented urgently
**2 ≤ R ≥3**	Corrective/improvement actions to program in the short term
**R = 1**	Improvement actions to program requiring immediate action

Specific assessments of particular risk factors, resulting from investigations, will be included in specific documents of the particular food supply chain.

This risk assessment is achievable for each phase of food supply chain in such a way as to provide a global overview of the nutritional analysis.

It is then necessary to add each value of risk for each step in the food supply chain, according to the following formula:

$$ {\displaystyle \sum R\left(pa,b,c\dots n+1\right)} $$

R is the risk value of the single phases. P a,b,c…n + 1 are the phases of the food production chain for at which it is necessary to perform a risk assessment.

Risk management depends on good science but it is not a scientific activity: it is an agency decision-making process that entails consideration of political, social, economic, medical and engineering information with risk-related information to develop, analyze, and compare regulatory options and to select the appropriate regulatory response to a potential health hazard.

It may need to consider alternative sets of assumptions that might lead to divergent estimates of risk; to address social, economic, ecological, and ethical outcomes as well as the consequences for human health and safety; and to consider outcomes for particular populations in addition to risks to whole populations, maximally exposed individuals, or other standard affected groups.

The important role of food and nutrition in public health is increasingly being recognized as crucial for its potential impact on health-related quality of life and the economy, both at the societal and individual levels. Increasing epidemiological and scientific evidence demonstrates clearly the links between food and health maintenance/disease development.

The basic principle underlying the HACCP system is that it is possible to identify potential hazards and defective practices at an early stage in a food operation. In Europe, the HACCP system is regulated by law to guarantee food safety and consumer health “from farm to fork”, taking into account the economic interdependence of intermediate on final uses [44].

For the total protection of consumer welfare, it is necessary to implement a number of actions, taking into account the entire supply chain and addressing the issue of quality not only through the health system certification, such as the HACCP system, but also by prolonging and preserving the presence of essential components of the nutritional and nutraceutical food, through a process of nutrient analysis.

The NACCP process takes the entire food chain from farm to consumer into consideration, so at every point of the chain it is necessary to implement a strict monitoring in order to guarantee total nutrition quality.

The steps that must be regulated in the food supply chain concern: *i*) primary production (breeding or agriculture); *ii*) production technology; *iii*) storage and distribution; *iv*) storage and retail; *v*) sale and catering; *vi*) home consumption.

## Conclusions

Providing the consumer with safe food is conditioned by different life styles, food habits, or conceptions of individual responsibility particularly in the age of globalization, and represents a constant challenge in developed and developing countries. To ensure food safety and nutritional quality throughout the entire food supply chain, “from field to consumer”, establishing a new concept capable of consolidating these safety and quality benefits at the level of consumer health was necessary.

We present the NACCP process for the first time, defined as a set of procedures, decisions and protocols that allow maintenance of a high standard of quality throughout the entire food production chain, until the verification of the effect on human health.

The NACCP process encompasses the concept of GNP, which include all the “ good manufacturing practices ” of each stage of the production chain, from the primary producer and to domestic food processing methods, in order to maintain nutritional quality in so much as possible. Starting from this global vision, NACCP defines a number of principles by which GNP are maintained, but at the same time posits specific operations employed in the analytical identification of a particular nutrient, which may potentially contribute to the welfare of the consumer.

The main purpose of all good practices in the food safety circle is to provide consumer with safe, healthy, and high quality food. In this contest we offer a novel approach permitting tight integration of all good practices relevant to GNP [33].

NACCP uses analytical methods for the tracking of nutrients, identified as biomarkers of nutritional quality. Moreover, the NACCP process takes into account the scientific substantiation of the functional effect of food.

Preventing damage to the nutrient is fundamental to the NACCP process, as it may go on to play an important role in the health of the consumer.

The study of the effects on human health and the monitoring of each stage of the supply chain, in order to preserve the nutritional quality of food, are dealt with in the context of the NACCP process. Moreover, the NACCP process lays down the guidelines for scientifically justifying nutritional claims, as requested by Regulation EU 1924/2006 for health claims.

The contribution of any food toward an individual’s well-being is as complex as the individual themselves. Further research should evaluate the proposed concepts under real working conditions.
